# Electronic Health Record Implementation: A Review of Resources and Tools

**DOI:** 10.7759/cureus.5649

**Published:** 2019-09-13

**Authors:** Roboam R Aguirre, Orlando Suarez, Mailenys Fuentes, Marcos A Sanchez-Gonzalez

**Affiliations:** 1 Clinical and Translational Research, Larkin Community Hospital, Miami, USA; 2 Miscellaneous, Larkin Community Hospital, Miami, USA; 3 Cardiology, Larkin Community Hospital, Miami, USA

**Keywords:** electronic health record, medical record, ehr, emr, ehr implementation, electronic health record implementation, electronic medical record implementation, medical record implementation

## Abstract

Implementing an electronic health record (EHR) can be a difficult task to take on and planning the process is of utmost importance to minimize errors. Evaluating the selection criteria and implementation plan of an EHR system, intending interoperability, confidentiality, availability, and integrity of the patient health information data, while ensuring timely, accurate, and regulatory compliant generation of reports is a critical task.

This article discusses the selection and implementation plan that will primarily consist of assessing existing institutional workflows for each department, and it outlines the necessities and inclinations of the institution to include in the EHR system for the organization to function properly. Resources and tools are included to assist in the selection of the product as well as ideas on how to train staff and evaluate staff readiness. Regulatory requirements are also included for consideration during the initial process.

EHR increases the logistic productivity of workflows and offers a safer way to care for patients. To ensure efficiency, there is a series of steps the provider’s staff must follow to ensure proper implementation and handling of the EHR system. Before using the implemented EHR, it is recommended to have a testing protocol in place to ensure areas of possible staff confusion are identified and controlled. Using a proper implementation strategy for a new EHR system can facilitate success, minimize delays, and increase health care worker’s satisfaction and decrease the chances of usability being compromised.

## Introduction and background

While searching the internet for literature related to the selection and implementing of electronic health record (EHR) systems, it was observed that several publications were addressing a specific aspect or a few aspects of the selection and implementation. However, there were very minimal to no publications that include most, if not all of the required aspects from selection, regulatory, to implementation and post-implementation of the EHR system in one source. The authors list a series of recommendations and tools for use. All of these tools to facilitate the successful EHR system implementation process are in one single source.

## Review

EHR Implementation: resources and tools

Implementation of EHR systems has become a requirement in the United States. The Centers for Medicare and Medicaid Services (CMS) have lead this transition with financial incentives for healthcare providers and healthcare organizations, name "providers" in this paper. However, not implementing the EHR will bring financial penalties for non-compliance. Many providers have been using a legacy (current/old) system as an EHR. The EHRs are mostly hybrid and some providers have service-lines or departments with computers that do not have an informatics system build for their use. Neither they have systems that can interoperate with the computers from the rest of the organization; an example can be the pharmacy or laboratory departments.

Some providers may have certain departments with an informatics system integrated such as the lab or billing, but the reporting capabilities are not sufficient to meet the needs of the business and the accreditation requirements and thus reports are manually created. For these clinical and administrative healthcare informatics problems, providers give priority to the implementation of a certified EHR and allocate a budget for the acquisition and adoption of the healthcare informatics system: EHR.

The legacy EHR may meet some of the CMS Meaningful Use requirements but may not be fully ready to meet stage three of the meaningful use requirement. The areas needed for the legacy system to be fully compliant are for the EHR to be able to generate and transmit discharge prescriptions and provide patients with electronic access to their EHR [1].

In learning about certified EHR options, one has to also consider the cost of the implementation and adoption. Some healthcare workers will be pulled out from their daily responsibilities to attend to the needs of the change through EHR implementation. In searching for certified EHRs, the organization looked at these categories to evaluate the new system the usability, functionality, utility, and cost. The usability and functionality category were selected to test different routes of access while documenting and characterizing representations of specific data required for information. Utility was selected because the organization needed to evaluate how accurately and completely the health record will present diagnostic and therapeutic information [2].

Case study

At a 260 bed hospital, the selected EHR certified product [3] for evaluation was Medhost EHRs; Cerner [4] was also considered. MEDHost Enterprise 2019 consolidates clinical, electronic prescription, patient education, and financials in one product [5]. The key features evaluated on MedHost were the computerized provider order entry (CPOE) system, clinical decision support system (CDSS), drug-drug allergy interaction checks, patient-specific education resources, data portability, authentication, access, control, and authorization among others.

The selected EHR met most of the specifications per criteria selected by the hospital and was the most economical software and still met the requirements. The implementation of the new EHR, will mitigate patient safety events from occurring such as medication errors due to pulling the wrong medication for a patient, minimization of risk of loss of patient health information due to the security systems that will be implemented such as specific user access to information per employee role and username and password, and compliance with HIPAA rules and accrediting and licensure agencies. Also, it will provide patients access to their health information, which meets meaningful use criteria.

The implementation process will first consist of performing an evaluation of the current organization’ s workflows for every department in two to three days, define the needs and preferences of the organization to have in the EHR for the hospital to function planned into one to three months, including hardware needs, deployment of selection and training of super users, to take about one week, and then plan the implementation and staff education to go live in about one more month. The plan for complete implementation roll out from go-live day to full adoption will be between six and eight months.

Testing the EHR

Testing the implemented EHR ensures that every system in place is put through its paces to ensure data tables and files are loading properly, data collected are processed and store correctly. In addition, it ensures that the system interfaces work as intended, that the workflows have been adjusted appropriately, that alerts are properly triggered and responding correctly, that the reports are generated accurately and completely and that the security system is also checked to ensure it is correct.

This paper offers a list of objectives for the test, set a scope of the test, establishing a testing strategy, establishing the environment requirements, create a test schedule, establishing a control procedure, identify functions that will be tested, creating a list of deliverables, and identifying risks.

Testing scope and environment required

In preparation for testing, the scope of the test will be defined as the testing for infrastructural readiness, application configuration readiness, and training readiness. These testing will include the evaluation of workstations, printers, servers, and wireless devices and related security measures. This part of the plan will include testing all workflows, procedures, and areas of the clinical record design. Training will also be included in this part of the testing to evaluate staff readiness in the use of the system before “go-live.”

The testing will be performed using a testing environment with actual patient data but on a separate section of the database that is not in production. Wright, Aaron, and Sittig [6] recommended to also test the system in a production environment for the system to mimic how it will behave when the system is live. After the system goes live, an ongoing regression testing assists in identifying that the system has not created new issues and that any new problems are identified and addressed.

Objectives for the test

Developing testing objectives such as the ones listed herein will help ensure that the system is working properly and producing the correct results. One of the objectives of the testing is to test the unit and functionality. This testing ensures that the major functions perform as required, that the customizations work as requested, that the screens appear as intended and screen functions properly, that there are no spelling errors, and that the content is appropriate. A second objective is to test the system to ensure that workflows send and receive data properly between the systems, that the interfaces between applications move data correctly and completely, that users gain access per assigned privileges, and that the data are processed accurately, in graphs, stables, claims, summaries, and it populates correct data in registries, reporting warehouses, etc.

The third objective will be testing the integrated system to ensure all system components work together, ensure workflows reflect the processes and workflows of the organization, that it follows policy and procedures, and that the system works with all mobile devices and with all of the devices and system capabilities such as voice recognition and speech commands, etc. The fourth objective will be to test for performance and stress to ensure response times process interactions within the system timely and within acceptable limits, evaluate system usage peak loads for accessibility and timely process of requested data, and timely generation of reports and evaluation of time for data dumps on system performance.

Testing strategy and test schedule

The strategy to test the system includes a pre and post-go-live system testing, a patient communication guideline to include expected downtime, staff scheduling and required overtime or temporary staff, modification of appointments and scheduling, reporting processes for system and project evaluation, communication tools (boards, etc.), network speed and reliability checks, and data backup processes. The purpose of performing testing is to ensure the system works and performs as expected [7-8].

During system testing, one needs to ensure that the team conducts unit testing (i.e., single module), conducts integration testing (i.e., interaction between two or more modules), conducts interface testing (i.e., interaction between systems), conduct system stress or load testing, ensures testing plans cover different scenarios and situations, contingency planning by developing a disaster recovery plan, testing the ability to restore system from backups prior to go-live, ensuring that the system backup plan in place and running and arrange for regularly scheduled pick up and off-site storage of backups [9]. During testing, day one will begin with testing for data availability to ensure that data is accessible and usable upon demand by authorized individuals, test for data quality and integrity to ensure the data and information is accurate and created appropriately and have not been altered or destroyed in an unauthorized manner, test for data confidentiality to ensure data and information is only available and disclosed to authorized users and processes, during testing day two, testing will be to test for complete and correct electronic health record use to ensure its features and functionality are implemented and used as intended, and will also test for system usability to ensure its features and functionality are designed and implemented so that it can be used effectively, efficiently, and to the satisfaction of the user. During testing day three will test again to monitor and improve patient safety to ensure that an ongoing quality assurance and performance improvement mechanisms are in place to monitor, detect, and report on the safety and safe use of electronic health records [10].

Identified risks

The risks associated with the testing and implementation of the new EHR system are those related to not achieving the objectives set for the EHR implementation. Having data that is incomplete, missing or misleading, open or incomplete patient orders, procedures and policies that are ineffective, failure to follow up abnormal test results, confusing one patient with another, reliance upon inaccurate or incomplete patient data, intentionally or accidentally subverting Clinical Decision Systems (CDS), automatic discontinuation of a prescription, data aggregation leading to erroneous data reporting, and prolonged EHR downtime among other legal related mandate risks.

Functions that will be tested, list of deliverables, and control procedures

The testing plan checklist from Health IT in Table 1 [11] below, is a great tool to use to ensure functions and components are tested, identify all deliverables, and to control all items to be identified and addressed with documented findings and plans to solve issues.

**Table 1:** The National Learning Consortium Testing Plan checklist

Test	Components	Date	Responsibility	Accepted
Unit & Functional Testing	Each major function performs as specified in user manual.			
	Design changes/customizations are present & work as requested. Document all changes for reference.			
	Screens appear as expected (content and placement of fields, codes, drop down menus, and messages).			
	No spelling errors or color changes. Readable icons.			
	Appropriate representation of content can be printed if necessary for legal purposes.			
	Entries that have been corrected and their corrections are both displayed accurately.			
	Fields edits (e.g., valid values, options, defaults) function as expected.			
	Alerts and clinical decision support provides appropriate reminders and prompts. Use scripts to test various scenarios.			
System Testing	Workflows send and/or receive data properly between systems (e.g., between EHR and pharmacy or billing, PMS messages and EHR). Use scripts to test various scenarios.			
	Interfaces between applications move data correctly and completely. Test both sending and receiving when interfaces are bi-directional.			
	Connectivity with external organizations is accurate and complete as authorized (e.g., portal access to/from hospital/clinic, continuity of care record to referrals, personal health records for patients, disease management to/from health plan).			
	System access is appropriate per assigned privileges. Test attempts to gain access when not authorized.			
	Data are processed accurately, in graphs, tables, claims, client summaries, reports, etc.			
	Data correctly populate registries, reporting warehouses, etc.			
Integrated Testing (simulates live environment)	Ensure all system components that share data or depend on other components work together properly.			
	Ensure that workflows reflect actual new processes and workflows.			
	Ensure that usage is defined in and follows policies and procedures. Reinforce training as applicable.			
	Ensure that help desk, support personnel, and other aids function properly.			
	Ensure that EHR works with all forms of human-computer interface devices and modalities being used (e.g., tablets, PDAs, voice recognition, and speech commands as applicable).			
	Attempt to break the system by testing mission critical and high risk functions, such as situations requiring exception logic (e.g., overrides to clinical decision support), handoffs from one process to another, and when you may have a series of events over a period of time (e.g., assessments performed at designated intervals).			
Performance & Stress Testing	Measure response times for key transactions or interactions with the system, and assure they are within acceptable limits, which may be defined in the contract.			
	Simulate an extremely high volume of activity on the system such as would exceed anticipated peak loads of system usage.			
	Measure the time it takes to generate reports and data dumps, and the impact on system performance.			

The EHR roll out

The strategy to roll out an electronic health record will be that of planning each step of the execution of the project until go-live and after by monitoring and controlling the effectiveness of the system. It is important to consider each step of the execution to ensure a smooth transition of the system and increase staff satisfaction with the use of the system for usability purposes. To implement the EHR, the implementation team must consider the approach to the implementation and the strategy of the implementation.

There are two different types of approaches to launching the electronic health record the Immediate Approach and the Incremental Approach. The Immediate Approach has the advantage of minimizing the time spent managing both a paper record and the new electronic system simultaneously, it can be disruptive and small system glitches can be amplified [12] The Incremental Approach has two different formats, one is by turning on certain functions in a step-wise approach and the other is by implementing the EHR in certain sites or departments and slowly roll out to the rest of the organization, learning and tweaking the process along the way; Table 2 below shows the different implementation approaches that can be used (Hodgkins, 2015) [12].

Upon the decision of the launching approach, the implementation strategy can then be considered based on the approach. Table 3 below shows the different implementation strategies that can be used based on the approach (Hodgkins, 2015) [12].

**Table 2:** The advantages and challenges of each electronic health record (EHR) implementation approach: immediate and incremental

APPROACH	
	IMMEDIATE	INCREMENTAL
ADVANTAGES	Eliminates confusion among staff since all administrative and clinical tasks will be completed electronically	Reduces productivity loss due to operational and workflow challenges
	Benefits are realized quickly	Issues are easier to resolve because they are isolated from other EHR modules or functions
		Allows staff to gradually learn and master the capabilities of the system
CHALLENGES	Requires significant resources and staff support	Requires to strictly follow a work plan to keep implementation phases on track
		Requires close attention to hybrid processes because not all tasks are completed electronically
		Requires awareness of the different functions that are being launched on different dates

**Table 3:** Strategies for immediate and incremental electronic health record (EHR) implementation

APPROACH	
STAKEHOLDERS	IMMEDIATE	INCREMENTAL
PHYSICIANS and STAFF	Mobilize all physicians and staff to use the EHR on the first day of launch. This allows all users to access implementation resources and enables all users to gain proficiency in the EHR at the same time	Train physicians and staff with the basic EHR functions and focus on optimization after the launch is complete. This allows physicians and staff to acclimate to their new system before bringing trainers back to provide additional/ supplemental education
PATIENTS		Establish mentorship program that enables staff with similar roles to share their knowledge and experience with the system, rapidly increasing the level of EHR proficiency in the practice e.g., physician super user teaches other physicians, experience nurses shadow other clinical staff.
		Start with enthusiastic and prepared physicians and staff using the EHR the first week and gradually increase the number of physicians and staff using the system
	Use the EHR for all patients in the facility. This approach can minimize variation of protocols used for different patients and appoint types	Use the EHR according to visit type, e.g., new patients, patients with appointments, ER, observation, inpatients, OR, etc.
		Use the EHR according to number of patients visits per day per census.

Implementation strategy: identification and selection

Based on the identified approach and strategies for electronic health record implementation presented, the implementation team has decided to use the incremental approach and strategy to improve physician and staff satisfaction, identify and gaps or system glitches that can appear and have the opportunity to correct them before full implementation, and reduce productivity loss due to implementation. If on the other hand, the team should have decided to use the Immediate approach, there was a consensus within the implementation team that the staff and physicians may not be satisfied with the EHR and thus usability will be compromised as well as decrease in productivity due to all staff expending time on learning and running into system issues and possibly delaying the implementation and proper use of the electronic system.

Recovery plan

When all implementation has been completed and the EHR system begins to be used, the question that remains to be answered is that of what will happen if the system fails for any reason, including not having electrical power. A recovery plan will be recommended to be considered and be in place to ensure that there is continuity of the service even though the electronic health record may not be available. Recommendations [8,13-15] to consider ensuring the purchased EHR system includes all necessary hardware, software, and utilities to support system back-up and recovery processes. Ensure that the system includes “redundant processors that contain copies of data files from the active EHR system to be quickly switched to the redundant system to continue operations” [13].

The recovery and back-up plan of the EHR is not only a best practice to include in the implementation of the new system, but it is a requirement. The Health Insurance Portability and Accountability Act (HIPAA) mandates for providers to back-up their electronic health record system and have a plan in place in case it fails. The elements that the HIPAA rule has mandated to incorporate to ensure that electronic protected health information (PHI) is secured in case of emergencies or disasters are data back-up plan, disaster recovery plan, emergency mode operation plan, testing and revision of procedures, and applications and data criticality analysis [8,16-17]. Table 4 shows the recommended processes to consider having a back-up plan in place (Code of Federal Regulations, 2007; Contingency planning, 2011; O’Dowd, 2018; Sahi, Lai, & Li, 2016; Tate, 2015; U.S. Department of Health & Human Services. 2013) [8,14-17].

**Table 4:** Processes to consider in backing up electronic health record (EHR) systems

Process Step	Purpose
Test System Back-up and Recovery processes Periodically	Ensures documented instructions are accessible and easily understood
Secure System effectively Secure System effectively	Ensures that computer system and data are secure by securing equipment in a secure room or iCloud with the appropriate air conditioning, fire protection, unlimited power supply, surge protection and other equipment and controls. Maintain integrity of the records
Develop interim Clinical and operations processes	Using manual methods to support patient care and practice operations should be implemented in case the EHR system is not available
Periodically Test	Test periodically for staff to understand what needs to be done and to check the maintenance of the integrity of records
Periodically Update Interim Clinical and Operations Processes	Test periodically for staff to be prepared to perform their tasks using manual forms and processes and updating them as needed or required. Keep exact copies of patient information
Assign a “Contingency coordinator”	Assigning a Contingency Coordinator and an alternate is important as this person will provide communication, coordination, and control necessary to get the system back to full operations as quickly as possible and maintain the back-up plan and processes updated

Training

A recommendation for training is to complete a user skills assessment and training needs before implementation. A user skills assessment evaluates the healthcare worker’s basic computer literacy and skills. This assessment could be considered as a competency demonstration and training can be provided as needed. There are many healthcare workers that a highly proficient in the use of technology such as computers, however, there is segment of the healthcare workforce that may need basic computer training such as how to use the mouse, navigate on a screen with the use of a scroll up and down, identification of tools, etc. The skills assessment is to be done before implementation to ensure skills are build up and ready by the time go-live happens.

Training strategy

The training strategy can be that of selecting super-users per each of the Health Information Systems such as Billing, Quality, CPOE, Nursing, etc. Super-user training should be considered to be happening before implementation to become familiarize with the system, feel comfortable teaching their part of the system, and in case the selected staff prefers not to be included. Consideration of having two super-users per health information system be trained is recommended in case the primary super-user goes on vacation or leaves the organization, this way there is always someone trained available to staff. The training approach could be that of a peer-to-peer approach as it facilitates training as peers communicate and speak the same language of the people they are training. Other teaching methods can also be included at the same time such as classroom-based training, e-learning modules, hands-on learning methods, one-on-one, and training staff only on the EHR areas they are going to use to avoid confusion and expedite the training and implementation [18].

Training should be considered to be performed throughout implementation and after go-live to ensure users feel competent and comfortable with the use of the new EHR system. Training session length times may vary from 1 hour to 8 hours especially for physicians who may need longer times. Training for staff should be considered to begin 2-3 weeks before implementation and go-live for basic skills and then classroom sessions, followed by e-learning, hands-on, and one-on-one training at different days and times of the day. Re-training should continue for optimization after go-live for 6 to 10 weeks. After the 10 weeks, re-assessment status should be done to evaluate the need for more training.

## Conclusions

EHRs not only provide a means of organizational efficiency but provide a safer way to care for patients and the required means to meet regulatory standards. The implementation process is an important step to consider when adopting an EHR in healthcare organizations. Selecting and including the appropriate strategy can facilitate success and minimize delays in the system rollout. Becoming familiarized with the approach and understanding the needs of the organization are detrimental steps to the success of the implementation. Using the best approach, strategy, back-up system and training increases healthcare worker’s satisfaction and exponentially decreases the chance of usability been compromised.
